# Short‐term thermal acclimation modulates predator functional response

**DOI:** 10.1002/ece3.8631

**Published:** 2022-02-17

**Authors:** Arnaud Sentis, Lukas Veselý, Marek Let, Martin Musil, Viktoriia Malinovska, Antonín Kouba

**Affiliations:** ^1^ INRAE Aix Marseille University UMR RECOVER Aix‐en‐Provence France; ^2^ Faculty of Fisheries and Protection of Waters South Bohemian Research Center of Aquaculture and Biodiversity of Hydrocenoses University of South Bohemia in České Budějovice Vodňany Czech Republic

**Keywords:** acclimation, functional response, metabolic theory, temperature

## Abstract

Phenotypic plastic responses to temperature can modulate the kinetic effects of temperature on biological rates and traits and thus play an important role for species adaptation to climate change. However, there is little information on how these plastic responses to temperature can influence trophic interactions. Here, we conducted an experiment using marbled crayfish and their water louse prey to investigate how short‐term thermal acclimation at two temperatures (16 and 24°C) modulates the predator functional response. We found that both functional response parameters (search rate and handling time) differed between the two experimental temperatures. However, the sign and magnitudes of these differences strongly depended on acclimation time. Acclimation to 16°C increased handling time and search rate whereas acclimation to 24°C leads to the opposite effects with shorter handling time and lower search rate for acclimated predators. Moreover, the strength of these effects increased with acclimation time so that the differences in search rate and handing time between the two temperatures were reversed between the treatment without acclimation and after 24 h of acclimation. Overall, we found that the magnitude of the acclimation effects can be as strong as the direct kinetic effects of temperature. Our study highlights the importance of taking into account short‐term thermal plasticity to improve our understanding of the potential consequences of global warming on species interactions.

## Graphical abstract

Temperature effect on feeding rate depends on acclimation temperature and duration. Acclimation to cool temperatures decreases predation rate whereas acclimation to warmer temperature leads to opposite effects. Moreover, the magnitude of the acclimation effects can be as strong as the direct kinetic effects of temperature.

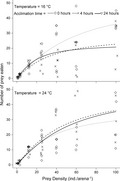

## 1 INTRODUCTION

The consequences of global warming on aquatic and terrestrial biodiversity are expected to increase during the next decades (IPCC, 2018). Warming increases the metabolic rate of ectotherm organisms and can thus be a stress factor when the metabolic demand increases faster with temperature than energy gains (Sentis et al., 2012, 2017; Vucic‐Pestic et al., 2011). Organisms can adapt to temperature change through dispersal, genetic changes across generations or plastic responses (Boukal et al., 2019; Parmesan, 2006; Parmesan et al., 1999). While the two first mechanisms have been studied in detail, the third one (plastic response) has received less attention, although the literature on this topic is currently growing fast. Many species exhibit plastic responses and these plastic responses are suspected to play an important role in the response of organisms to climate change (Sandoval‐Castillo et al., 2020). Extended exposure to warmer temperature can lead to thermal acclimation causing physiological changes in an organism that can affect the shape and position of thermal performance curves (Schulte et al., 2011). For instance, thermal acclimation can increase the critical thermal maximum or optimal performance temperature of a biological trait (Rohr et al., 2018; Sinclair et al., 2016), such as metabolism, behavior, or immunity (Dietz & Somero, 1992; Raffel et al., 2013; Terblanche et al., 2007). In line with the beneficial acclimation hypothesis (Wilson & Franklin, 2002), these changes in the shape and position of thermal performance curves can enhance species tolerance to higher temperatures by reducing the metabolic costs of living at high temperatures or increasing foraging efficiency under warming (DeWitt et al., 1998; Donelson et al., 2011; Sentis et al., 2015). However, the consequence of plastic responses to temperature for species interactions remains poorly investigated (but see Sentis et al., 2015). This is an important limitation because species interactions govern the fluxes of energy and materials and are thus crucial for community dynamics and ecosystem functions.

Altered temperature regimes can affect individuals, populations, and communities directly and indirectly in multiple ways (Boukal et al., 2019). Temperature directly alters metabolism and other physiological rates (Brown et al., 2004), and hence modifies behavioral and life‐history traits of individuals (Abram et al., 2017; Dell et al., 2011). These direct effects translate into altered species interactions, which in turn determine community structure and dynamics (Rosenblatt & Schmitz, 2016; Sentis et al., 2017; Uszko et al., 2017) and provide ecological and evolutionary feedbacks to the individuals (Stoks et al., 2013, 2015). Previous studies showed that warming can affect food‐web stability and structure by modifying the strength of trophic interactions (Binzer et al., 2012; Kratina et al., 2012; O’Connor, 2009; Sentis et al., 2014). Most consumer species become less efficient at processing matter and energy at warmer temperatures as their metabolic rates often increase faster with temperature than their feeding rates (Archer et al., 2019; Iles, 2014; Vucic‐Pestic et al., 2011). This reduction of energetic efficiency as well as lower interaction strength stabilizes food‐web dynamics by reducing population fluctuations (Gilbert et al., 2014; Rip & McCann, 2011). However, this reduction in energetic efficiency can also lead to negative population growth rate and population extinctions in the longer term if organisms cannot acquire enough energy to cover metabolic losses.

While these previous studies helped better understand the direct effect of temperature mediated by short‐term philological and behavioral responses to temperature, they did not address the potential impact of thermal acclimation on these interactions. A previous study by Sentis et al. (2015) showed that metabolic rate was not influenced by acclimation while acclimation to warmer temperature tended to increase the predator’s feeding rate. Overall, the performance of acclimated predators, measured as energy gains over metabolic losses, was higher than the performance of non‐acclimated ones (Sentis et al., 2015). They suggested that thermal acclimation could be important for trophic interactions. However, they acclimated the predators for several weeks at only two mild temperatures which limits the evaluation of the importance of short‐term acclimation to even higher or lower temperatures. Moreover, most previous studies considered relatively long‐term (several days or week) acclimation and the impact of short term (hours or a few days) remains largely unexplored. Given that the frequency of climatic variations and rapid temperature fluctuations are expected to increase with global warming (IPCC, 2013; Prats et al., 2020), short‐term acclimation might become even more important for species adaptation to fluctuating environments. Moreover, water levels of most lakes, pounds, and rivers are expected to be lower during summer (IPCC, 2013) which will also increase climatic variations as shallow water are prone to rapid temperature changes (Prats et al., 2020).

To fill this gap, we used crayfish and their isopod prey as a model system to test the effect of short‐term thermal acclimation on predator‐prey interactions. We acclimated predators for 0, 4, or 24 h at two experimental temperatures (16 and 24°C) and then measured their predation rate at different prey densities (i.e., their functional response). We expected that (i) predation rate should increase with temperature due to higher metabolic demand under warming (Boukal et al., 2019; Brown et al., 2004), (ii) acclimation to warmer temperature should exacerbate the direct effect of temperature on maximum predation rate to cope with the higher metabolic demand (Sentis et al., 2015), and (iii) the effect of acclimation should not differ for acclimation times 4 and 24 h as previous studies suggested that 4 h is enough to reset thermal clock for small ectotherm invertebrates (Sentis et al., 2014; Veselý et al., 2019b). Overall, our study aims at better understanding the role of thermal acclimation in trophic interactions.

## 2 MATERIAL AND METHODS

We conducted experiments using the marbled crayfish *Procambarus virginalis* Lyko, 2017 (Decapoda; Cambaridae) preying on the water louse *Asellus aquaticus* (Linnaeus, 1758) (Isopoda: Asellidae). Marbled crayfish is an invasive benthic actively searching omnivorous species spreading across freshwater ecosystems mainly in Europe (Patoka et al., 2016), but also emerging as a model species in various biological disciplines (Hossain et al., 2018; Vogt, 2011). Water louse is a common benthic detritivorous zoobenthos species inhabiting most of lentic and lotic habitats (Christensen, 1977).

Experiments were conducted at the Research Institute of Fish Culture and Hydrobiology in Vodňany, Czech Republic in September 2019. No specific permissions were required for the experiments, animal capture and maintenance in the laboratory. Crayfish were obtained from our own experimental culture. Water lice were collected using bottom kick nets in Příbramský stream (49.7063417°N, 14.0124939°E). We standardized crayfish size (mean total length: 37 ± 1.5 mm, measured from the tip of the rostrum to the posterior edge abdomen; wet weight: 0.45 ± 0.13 g measured by removing excessive water using an absorbing paper prior to weighing) and prey size using sieves with a mesh size of 3 mm.

Before the experiment, consumers and prey were maintained at 20°C and fed in excess with thawed sludge worms *Tubifex tubifex* (O. F. Müller, 1774) and detritus, respectively. Crayfish were kept at low densities (0.8 ind.L^−1^) in 50‐L aquaria with access to shelters (>1 per animal) to avoid competition and cannibalism. Prior to the experiment, crayfish were kept individually in 0.5‐L boxes and starved for 24 h. Prey were held in trays with aeration (256 × 48 × 18 cm, filled with 147.5 L of aged tap water). To test the effect of thermal acclimation on crayfish consumption rate, crayfish were maintained at given temperature 16 or 24°C for 0 (i.e., no acclimation), 4 or 24 h before the experiment. Acclimation temperatures and times were fully‐crossed resulting in 6 treatments (2 acclimation temperatures ×3 acclimation time). Prior to the experiment prey were held in buckets (20 L) at low density with aeration for 24 h at the experimental temperature (16 or 24°C) to avoid thermal shock. Experimental arenas (plastic boxes, 285 × 190 × 75 mm in size) were filled with 2 L of aged tap water and lined with a 1 cm layer of fine sand. All experiments were conducted over a 4‐h period under constant light conditions.

### 2.1 Consumer functional response

We quantified the functional response of marbled crayfish by measuring their feeding rate at 7 prey densities (1, 3, 12, 25, 40, 60, and 100 ind.arena^−1^) at each temperature (16 and 24°C) and acclimation time (0, 4, 24 h). The test temperatures were the same as the acclimation temperature (i.e., acclimation temperature and test temperature were not crossed) yielding 42 treatments (2 temperatures × 3 acclimation times × 7 prey densities). The temperature of 16°C is considered as sub‐optimal for warm‐water crayfishes, but still within the range for successful reproduction in the marbled crayfish (Seitz et al., 2005). 24°C is encountered in rather warmer habitats, although it may be more frequently recorded in the near future because of climate change. The chosen thermal acclimation time mimics rapid temperature changes observed in shallow ponds and lakes where temperature can fluctuate up to 8°C within a day (Prats et al., 2020, A. Sentis, unpublished data).

Prey were introduced in the experimental arenas 1 h before the experiment to minimize stress effects caused by manipulation. After this period, consumers were released gently into the arenas and the number of remaining prey in each arena was recorded after 4 h. For each combination of temperature and acclimation time, five replicates at each prey density were conducted. Moreover, five replicates without consumers were conducted for each prey acclimation time‐temperature‐density combination to control for potential prey background mortality.

When we counted the animals remaining in the experimental arenas, we distinguished the ones that survived from the dead (without visible marks of an attack) and killed (killed but not eaten prey) ones. In a previous study, we showed that crayfish can kill prey without eating them and that prey can also die in the presence of crayfish predators due to high stress levels (Veselý et al., 2017). In the present study, our objective was to assess the impact of temperature and thermal acclimation on short‐term interaction strength which represent the effect of a predator on the prey population density. We thus considered the total impact of crayfish on prey population by summing the numbers of dead, killed, and eaten prey (but see Figure S3 for results based on eaten prey only). The number of eaten prey was obtained by subtracting the number of dead and killed (but not eaten) prey from the initial prey density. Prey mortality in controls (i.e., treatment without consumers) was negligible (proportion dead: mean ± SD = 1.45 ± 2.44%) and data were thus not corrected for background prey mortality. In addition, prey killed but not eaten reach 35.82 ± 35.20% across treatments and densities.

For each temperature regime and acclimation time, a logistic regression between initial prey density (*N*_0_) and the proportion of prey eaten (*N*_e_/*N*_0_) was performed to identify the shape of the functional response:

(1) $$\frac{N_{e}}{N_{0}}=\frac{\exp\left(P_{0}+P_{1}N_{0}+P_{2}N_{0}^{2}+P_{3}N_{0}^{3}\right)}{1+\exp\left(P_{0}+P_{1}N_{0}+P_{2}N_{0}^{2}+P_{3}N_{0}^{3}\right)}$$

where *P*_0_, *P*_1_, *P*_2_, and *P*_3_ are the intercept, linear, quadratic, and cubic coefficients, respectively, estimated by the maximum likelihood (Juliano, 2001). If *P*_1_ < 0, the proportion of prey killed declines monotonically with the initial density of prey, matching a type II functional response. If *P*_1_ > 0 and *P*_2_ < 0, the proportion of prey killed is a unimodal function of prey density, corresponding to a type III functional response (Juliano, 2001). Our results indicated type II functional response for each consumer species at both temperatures (Table S1). We thus estimated functional response parameters using the type II Rogers random predator equation (Rogers, 1972) that accounts for prey depletion during the experiment:

(2) $$N_{e}=N_{0}\left(1‐\exp\left(‐a\left(t‐hN_{e}\right)\right)\right)$$

where *N_e_* is the number of prey eaten, *N*_0_ is the initial prey density per liter, and *a* is the consumer search rate (arena.day^−1^) corresponding to the volume of arena searched per day. It corresponds to the speed at which a predator moves through the landscape to find prey relative to prey velocity. *H* is the consumer handling time (day.prey^−1^) and *t* is the duration of experiment in days. Before fitting the Rogers model to our experimental data, we used Lambert *W* function to solve Eqn. 2 for *N_e_* (for further details see Bolker, 2008):

(3) $$N_{e}=N_{0}\frac{W\left(ahN_{0}e^{‐a\left(t‐hN_{0}\right)}\right)}{\mathrm{ah}}.$$

To determine if the search rate *a* and handling time *h* were influenced by temperature, acclimation time, and their interaction, we first bootstrapped (999 bootstraps) the raw consumption data using ‘frair_boot’ function from the ‘frair’ package (Pritchard, 2014) and refitted equation 3 using maximum likelihood estimations via ‘mle2’ function from the ‘bbmle’ package (Bolker, 2008) to obtain bootstrapped search rate and handling values for each of the nine temperature‐acclimation time treatments. We then used the bootstrapped values in two‐way ANOVAs with search rate or handling time as dependent variables and temperature, acclimation time, and their interactions as independent variables. For search rate or handling time, the final model was determined by sequential deletion of the least significant explanatory parameters (or interaction terms) from the full model. Parameter significance was evaluated using *F*‐tests from analysis of deviance. The final model included only parameters with significant *p*‐values. Post hoc Tukey tests were performed to investigate differences among treatments.

## 3 RESULT

**FIGURE 1:**
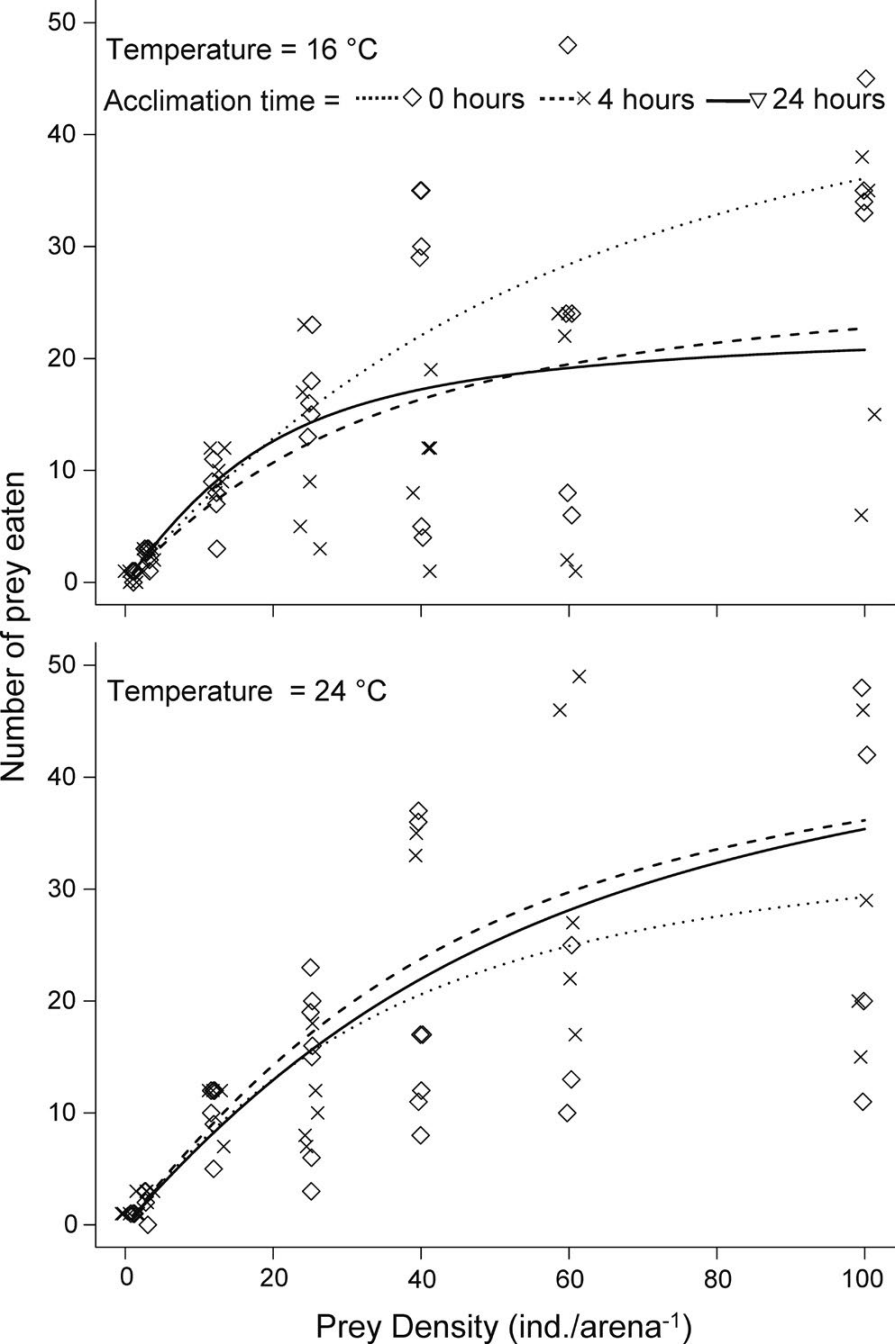
Functional responses of *Procambarus virginalis* feeding on *Asellus aquaticus* for different temperatures and acclimation times. Individual replicates (rhombus, cross, and inverted triangle) overlaid by prediction of the most parsimonious model (dotted, dashed,and solid line). Green rhombus and dashed line = 0 h acclimation time; grey cross and dotted line = 4 h acclimation time; purple inverted triangle and solid line = 24 h acclimation time. Predators and prey were maintained at 20°C before thermal acclimation started

For each consumer at each temperature and acclimation time, the relationship between prey density and the number of prey eaten was best described by a Holling type II functional response (Figure 1, Table S1). Predation rate first increased with prey density and then reached a plateau at high prey densities (Figure 1).

**FIGURE 2:**
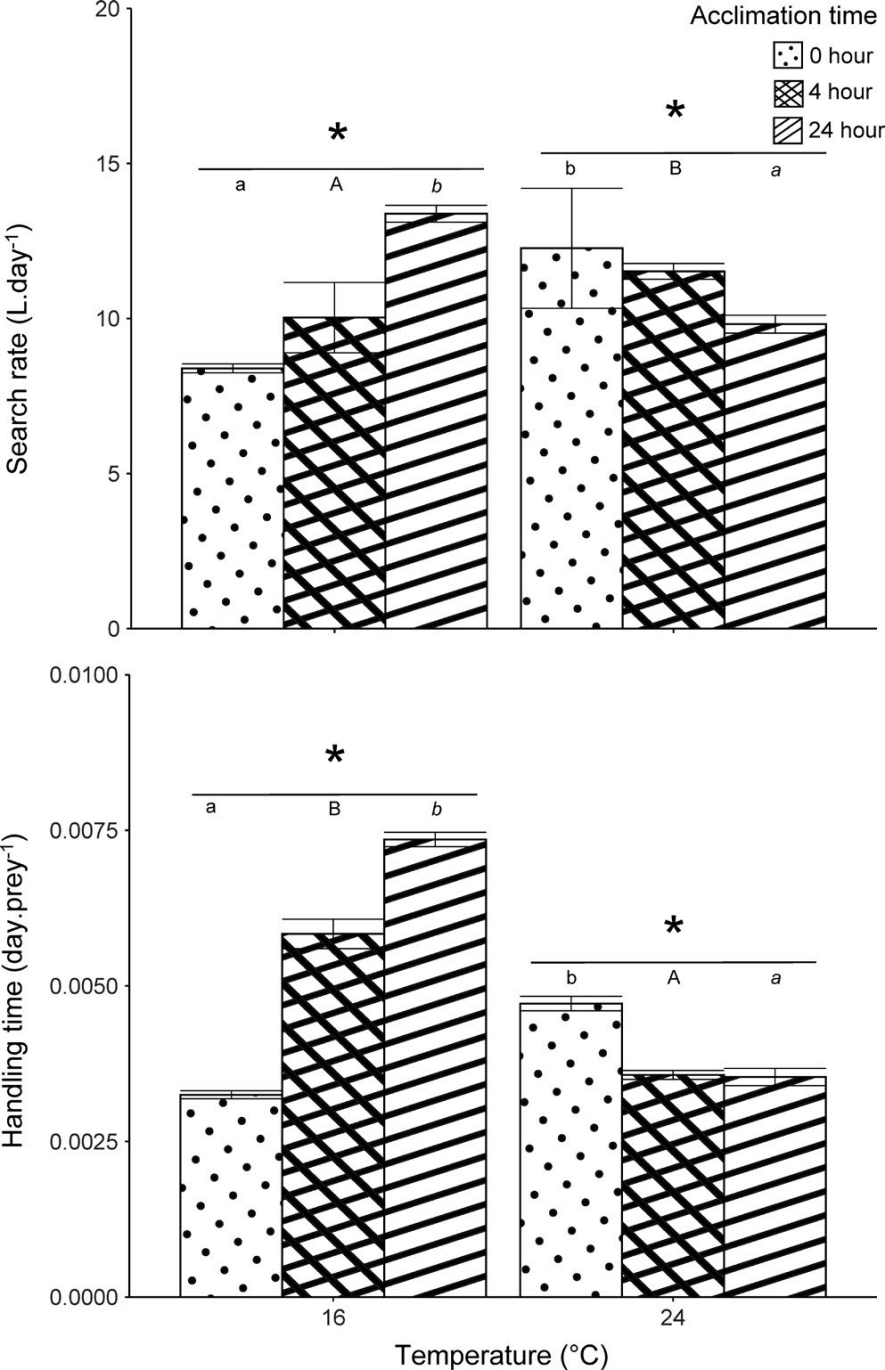
Estimated values (mean ± 95% CI) of search rate and handling time for *Procambarus virginalis* feeding on *Asellus aquaticus* for different temperatures and acclimation times. Significant differences (*p* < .05) between temperatures marked by asterisk. Different letters denote significant differences within acclimation time between temperatures (0 h = small letter, 4 h = capital letter, 24 h = small letter in italic). Predators and prey were maintained at 20°C before thermal acclimation started

We found that consumer search rate and handling time were affected by the interaction between temperature and acclimation time (*F*_4, 5994_ = 31.48, *p* ≤ .001; *F*_4, 5994_ = 220.96, *p* < .001; Figure 2 and Figure S1, respectively). Without acclimation, search rate was lower at 16°C compared to 20°C but this difference was non‐significant after 4 h of acclimation and then even reversed after 24 h of acclimation where search rate was higher at 16°C compared to 20°C (Figure 2). The effect of acclimation was thus different across the two temperatures. At 16°C, acclimation increased search rate indicated that the longer the predators were acclimated the more efficient they were at capturing prey. In contrast, we found the opposite pattern at 24°C were acclimation time decreased search rate (Figure 2 and Figure S2). These results indicate that both the time and the temperature at which predators are acclimated influence the predator search rate. When considering only the number of prey eaten instead of the total of prey killed, we found a similar pattern for the effect of increasing temperature with an increase in search rate without acclimation and after an acclimation of 4 h (Figure S3). However, the effect of acclimation was different as, for both temperatures, search rate was higher after 4 h of acclimation compared to 0 or 24 h of acclimation.

As for the search rate, the influence of acclimation on handling time depended on the temperature. At 16°C, handling time increased with acclimation whereas we found the opposite at 24°C. Moreover, acclimation reversed the impact of temperature on handling time as, without acclimation, handling time was longer at 24°C than at 16°C and, after 4 or 24 h of acclimation, we found the opposite where handling time was longer at 16°C compared to 24°C. When considering only the number of prey eaten instead of the total of prey killed, we found a similar pattern with, in absence of acclimation, handling time being longer at 24°C than at 16°C and, after 4 or 24 h of acclimation, the opposite with handling time being longer at 16°C compared to 24°C (Figure S3). As for the search rate, at both temperatures, handling time was higher after 4 h of acclimation compared to 0 or 24 h of acclimation.

## 4 DISCUSSION

Investigating the effect of temperature on trophic interactions is crucial to better understand and predict the impact of climate change on ecological communities (Boukal et al., 2019; Gilman et al., 2010). Previous studies highlighted the importance of evaluating the thermal dependencies of trophic interaction strength that are characterized in the short term by the predator functional response (Vasseur et al., 2014). However, the effects of thermal acclimation on functional response parameters remain largely unexplored (but see Sentis et al., 2015). Moreover, there is no consensus on how long animals should be acclimated before conducting the experiments. Acclimation time in previous functional response studies can range from a few hours (Novich et al., 2014; Sentis et al., 2012, 2014; Veselý et al., 2019a), to days (South & Dick, 2017; Wasserman et al., 2016), weeks (Haubrock et al., 2020; Sentis et al., 2015), or months (Daugaard et al., 2019; Gebauer et al., 2018) but it remains unclear how long is needed to reach the acclimated state and how long this state can last when temperature changes again. Moreover, most of the studies mentioned above used a single and fixed acclimation period before conducting functional response experiment which prevents testing how acclimation time influences predation rate.

Here, we showed that short‐term acclimation can have strong consequences on the predator functional response and modify conclusions about how temperature influences the predator search rate and handling time. This is of importance as there is a growing number of studies using empirically estimated parameters to predict the consequences of climate change on populations and communities (Archer et al., 2019). Here, we argue that this approach could be improved by accounting for plastic responses to temperature.

### 4.1 Acclimation reverts the direct effect of temperature on search rate

We found that, without acclimation, search rate was higher at 26°C compared to 16°C which is in line with previous studies reporting that search rate often increases with temperature across a range of non‐stressful temperatures (Englund et al., 2011; Rall et al., 2012; Sentis et al., 2012). Nevertheless, acclimation time had a strong influence on search rate. At 16°C, the longer the predators were acclimated the better they were at searching for prey. Interestingly, the pattern was opposite at 24°C with search rate decreasing with acclimation time. The results for acclimation to warm temperature are consistent with previous studies by Sentis et al. (2015) showing that predatory dragonfly larvae acclimated to warmer temperature had a low search rate that non‐acclimated ones. Overall, our results thus indicate that acclimation to cold temperatures increases the predator search rate while acclimation to warm temperature leads to the opposite effect. This result thus fails to support the beneficial acclimation hypothesis (Wilson & Franklin, 2002).

A significant number of empirical studies have rejected the generality of the beneficial acclimation hypothesis suggesting that acclimation responses are variables and their amount of variation probably depend on the trait investigated (Deere & Chown, 2006; Wilson & Franklin, 2002). Some aspects of physiology, like respiration rate or cell kinetics, may have strong predictable thermal responses, whereas behavioral traits including searching and killing prey are probably more variable. However, it is thus difficult to draw generalities as we only investigated one trait (predation rate) at two temperatures, and it would be important to confirm these findings across several experimental temperatures. Further studies are also needed to investigate the mechanism driving these opposite patterns, but it is possible that, at cold temperatures, predators need longer acclimation time to increase their physiological and search activity levels in a cold environment that slows physiological processes. In contrast, warm temperatures may be stressful and thus longer exposure to warmer temperature may result in a decrease in search activities as reported by Sentis et al. (2015). Whatever the mechanism underlying these patterns, our results imply that acclimation time can have a strong influence on the predator search rate. In some cases, the effect of acclimation was as strong as the effects of increasing temperature, which thus indicates that accounting for thermal acclimation can be very important when trying to predict the impact of climate change on species and their interactions.

### 4.2 Warming shortens handling time but acclimation to cold temperature leads to the opposite effect

Although we used only two test temperatures, we found that, after 4 or 24 h of acclimation, handling time was shorter at 24°C compared to 16°C which is line with results from previous functional response studies (Englund et al., 2011; Rall et al., 2012; Sentis et al., 2012). It implies that predators handle prey faster at warmer temperature and can thus consume more prey when prey density is high. Nevertheless, this pattern was strongly influenced by acclimation temperature. Acclimation to cold temperature (16 °C) increased handling time whereas acclimation to warmer temperature decreased it. The results at warmer temperature are consistent with the results of Sentis et al. (2015). Our results add to this previous study by showing that acclimation to cold temperature can lead to the opposite effect. The maximum feeding rate of most consumers is limited by digestion which implies that handling time mainly represents the time to digest a prey item as reported in previous studies (Jeschke et al., 2002; Sentis et al., 2013). It could thus be interesting to investigate how fast digestion processes can acclimate to temperature changes to better understand the mechanisms explaining the impact of thermal acclimation on handling time. Overall, we found that, when predators are acclimated, handling time is lower at warmer temperature while, when predator are not acclimated, handling time is higher at warmer temperature (Figure 2). This finding highlights the importance of taking into account acclimation as it can revert the effects of temperature on handling time which may have important implication for trophic interaction strength and top‐down control as the reverse of handling time (1/h) corresponds to the predator maximum feeding rate.

### 4.3 Potential impacts of acclimation on population dynamics

Weather and how the influence on thermal acclimation on the short‐term impact of predators on prey population translates into longer term population dynamics remain an open question. Based on our results, we can expect that, under constant temperature regime, acclimation to low temperature (i.e., 16°C) leads to high search rate but long handling time and thus a weaker ability of the predator to control high prey densities. In contrast, acclimation to high temperature leads to low search rate but shorter handling time and thus a stronger control of high prey densities. Under temperature fluctuations, the results would depend on how fast temperature fluctuates. In a scenario where temperature fluctuates daily between 16 and 24°C and predators thus have ~4 h to acclimate, then the average search rate should be lower than at 20°C constant while the average handling time should be similar to at 20°C constant. The predators may thus have a weaker control of prey density under daily temperature fluctuations when prey density is low. For a scenario where the time scale of fluctuations is longer and predators thus have ~24 h to acclimate, we found a similar scenario of lower average search rate and similar average handling time compared to constant 20°C.

Nevertheless, the impact of predators on prey populations also depends on the predator and prey population growth. Predator population growth depends on the balance between energy gain (through predation) and energy losses (metabolism). As metabolism increases exponentially with temperature and given that we did not observe an exponential increase in predation rate with temperature, we can expect lower predator densities at warmer temperatures and thus a weaker impact on prey populations. Moreover, our results indicate that the effects of acclimation can differ depending on if we consider only the number of prey eaten (and thus energy gain for the predator) or the total number of prey killed (and thus the impact of predator on prey populations). When considering only the number of prey eaten, the impact of acclimation on search rate was strongest after 4 h which suggests that longer acclimation time favors wasteful killing and should thus amplify the negative impacts of warming on predator populations. In turn, prey populations’ intrinsic growth rate and the ability of prey to escape predators may also depend on their thermal acclimation which we did not investigate in this study. This may be important as, even if search rate is higher for acclimated predators, consumption rate could remain unchanged if capture rate decreases with prey acclimation. We thus call for future studies to investigate thermal acclimation in both predator and prey as well as the consequences for longer term population’s dynamics or population abundance in the field (as in Archer et al., 2019). It would thus be interesting to conduct a similar experiment along a larger gradient of temperature and with more experimental temperatures to better determine the shape of the relationship between functional response parameters and temperature and how acclimation can modulate this shape. Moreover, it would be interesting to cross acclimation temperatures and test temperatures to determine the potential interactive effects of acclimation and test temperatures on functional response parameters. Finally, in the long term, thermal adaptation is more likely to occur and it can modify the shape and position of thermal performance curves (Knies et al., 2009) and thus modulate the impacts of warming on trophic interactions (Wang et al., 2020, 2021). Previous experimental studies showed that, in line with the hotter is better paradigm, warm‐adapted populations often perform better than cold‐adapted ones (Frazier et al., 2006), and that this can weaken the long‐term interaction strength between predators and prey. These studies together with our results thus indicate that short‐term acclamatory responses and longer term evolutionary responses may differ in their direction and intensity and thus impact trophic interactions in sometimes opposite directions.

## 5 CONCLUSION

Short‐term thermal acclimation can have a strong impact on trophic interactions which implies that (1) we should be careful when conducting predation experiments at different temperatures and should acclimate the organisms to the experimental temperatures prior to the experiment and (2) short‐term thermal acclimation could become even more important as climate warming increases temperature fluctuations and extremes and organisms will thus have to rely on short‐term thermal acclimation to cope with these rapid environmental changes.

## CONFLICT OF INTEREST

The authors declare that they have no conflicts of interest.

## AUTHOR CONTRIBUTIONS

**Arnaud Sentis:** Conceptualization (lead); Formal analysis (lead); Writing – original draft (lead); Writing – review & editing (lead). **Lukas Vesely:** Conceptualization (lead); Formal analysis (lead); Writing – original draft (lead); Writing – review & editing (lead). **Marek Let:** Investigation (equal). **Martin Musil:** Investigation (equal). **Victoria Malinovska:** Investigation (equal). **Antonín Kouba:** Conceptualization (equal); Funding acquisition (lead); Project administration (lead).

## Supporting information

Supplementary Material

## Data Availability

Underlying data are available on Dryad: https://doi.org/10.5061/dryad.mgqnk991n.
