# Supplementary material for: Short‐term thermal acclimation modulates predator functional response
Source: Ecol Evol. 2022 Feb 17;12(2):e8631. doi: 10.1002/ece3.8631 (PMC8855023; doi:10.1002/ece3.8631)
Supplement: Supplementary file 1 — Supplementary Material [file ECE3-12-e8631-s001.docx]

Supplementary information

**Table S1**. Logistic regression of the proportion of prey eaten by each acclimation time at 16 °C, and 24 °C. Value, standard error and P-value of intercept *P*_0,_ linear coefficient *P*_1_, quadratic coefficient *P*_2_, and cubic coefficient *P*_3_.

| **Acclimation time** |  |  | **16 °C** |  |  |  |  | **24 °C** |  |  |
| --- | --- | --- | --- | --- | --- | --- | --- | --- | --- | --- |
|  | Coefficient |  | Value | SE | P-value |  |  | Value | SE | P-value |
| 0 hours | *P*_0_ |  | -0.56 | 0.09 | <0.001 |  |  | -0.54 | 0.09 | <0.001 |
|  | *P*_1_ |  | -1.32 | 0.54 | 0.01 |  |  | -1.99 | 0.49 | <0.001 |
|  | *P*_2_ |  | 0.17 | 0.48 | 0.72 |  |  | 0.72 | 0.49 | 0.15 |
|  | *P*_3_ |  | 0.69 | 0.38 | 0.06 |  |  | 0.68 | 0.39 | 0.09 |
| 4 hours | *P*_0_ |  | -0.67 | 0.09 | <0.001 |  |  | -0.44 | 0.10 | <0.001 |
|  | *P*_1_ |  | -2.86 | 0.53 | <0.001 |  |  | -1.89 | 0.53 | <0.001 |
|  | *P*_2_ |  | 2.13 | 0.51 | <0.001 |  |  | 0.10 | 0.46 | 0.82 |
|  | *P*_3_ |  | -0.03 | 0.44 | 0.95 |  |  | -0.49 | 0.39 | 0.21 |
| 24 hours | *P*_0_ |  | -0.84 | 0.11 | <0.001 |  |  | -0.47 | 0.09 | <0.001 |
|  | *P*_1_ |  | -2.29 | 0.57 | <0.001 |  |  | -1.89 | 0.53 | <0.001 |
|  | *P*_2_ |  | 0.22 | 0.52 | 0.65 |  |  | 0.52 | 0.47 | 0.27 |
|  | *P*_3_ |  | 2.09 | 0.45 | <0.001 |  |  | -0.36 | 0.37 | 0.34 |


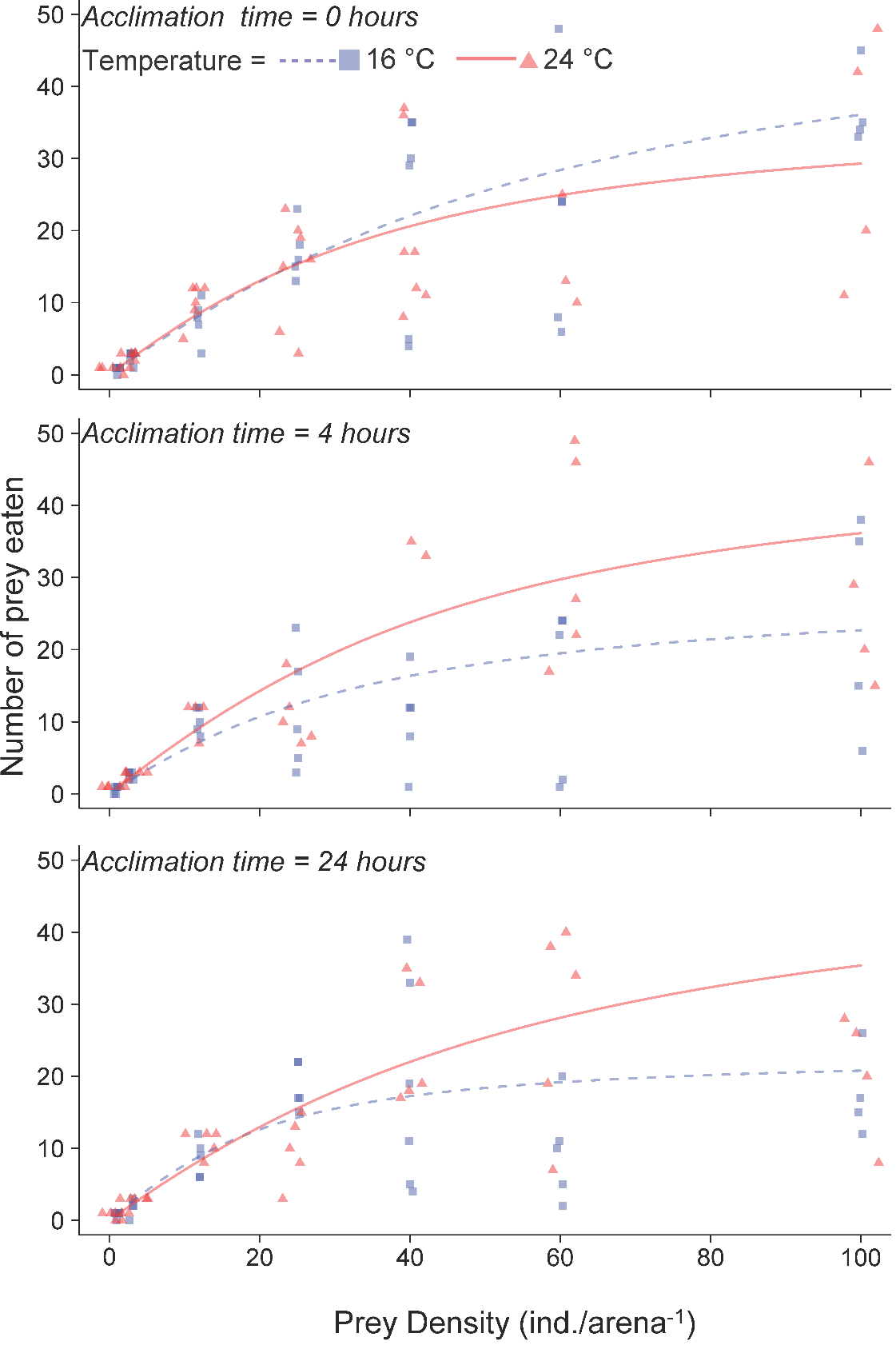


Figure S1. Functional responses from *Procambarus virginalis* feed on *Asellus aquaticus* in given temperature and acclimation time. Individual replicates (triangles and square) overlaid by prediction of the most parsimonious model (dashed and solid line). Blue square and dashed line = 16°C, red filled triangle, and solid line = 24 °C.


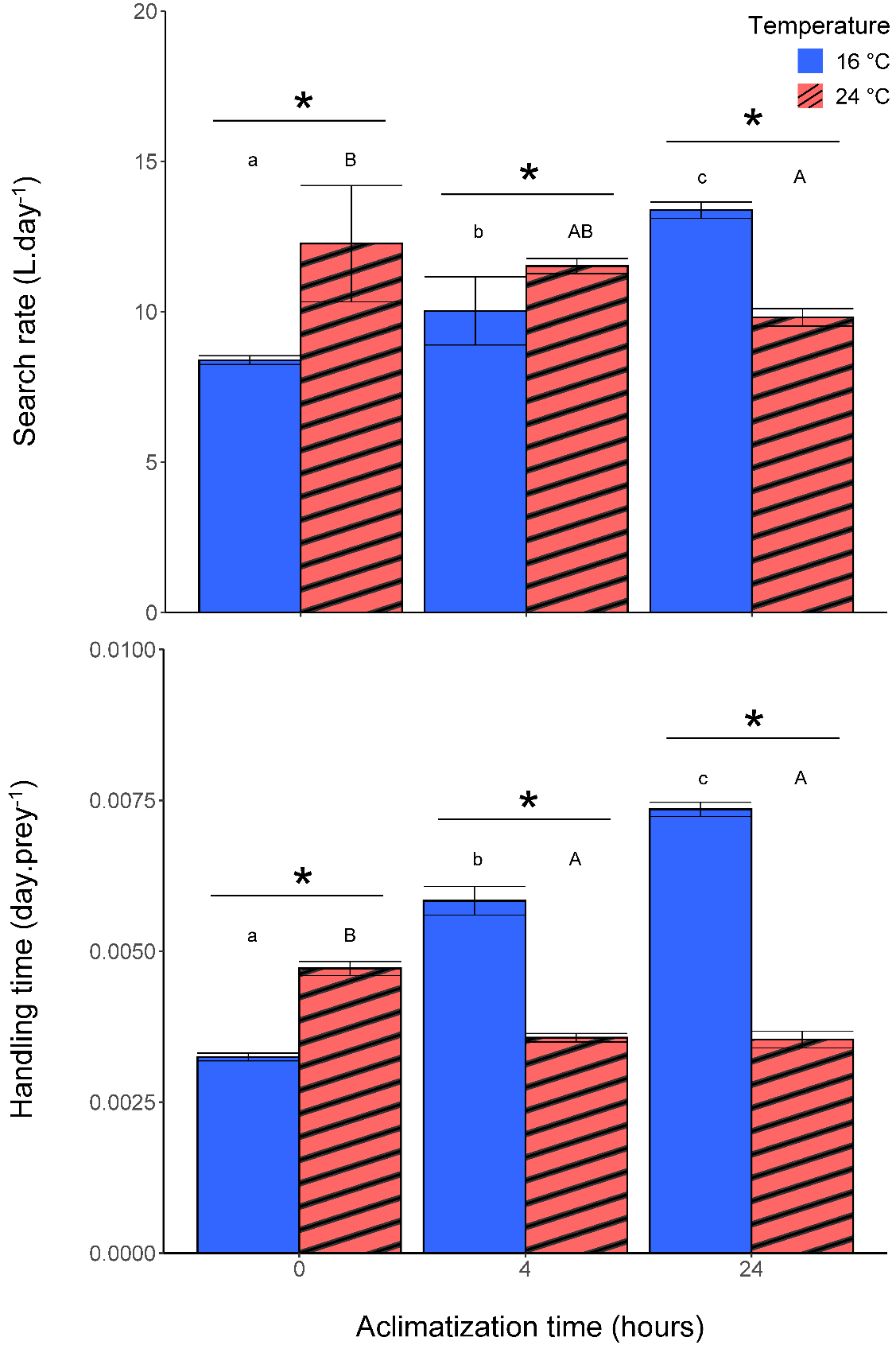


Figure S2. Estimated values (mean ± 95%) of search rate and handling time for *Procambarus virginalis* feeding on *Asellus aquaticus* for different temperatures and acclimation times. Significant differences (p < 0.05) between acclimation time marked by asterisk. Different letters denote significant differences within temperature between acclimation time (16 °C = small letter, 24 °C = capital letter Predators and prey were maintained at 20 °C before thermal acclimation started.


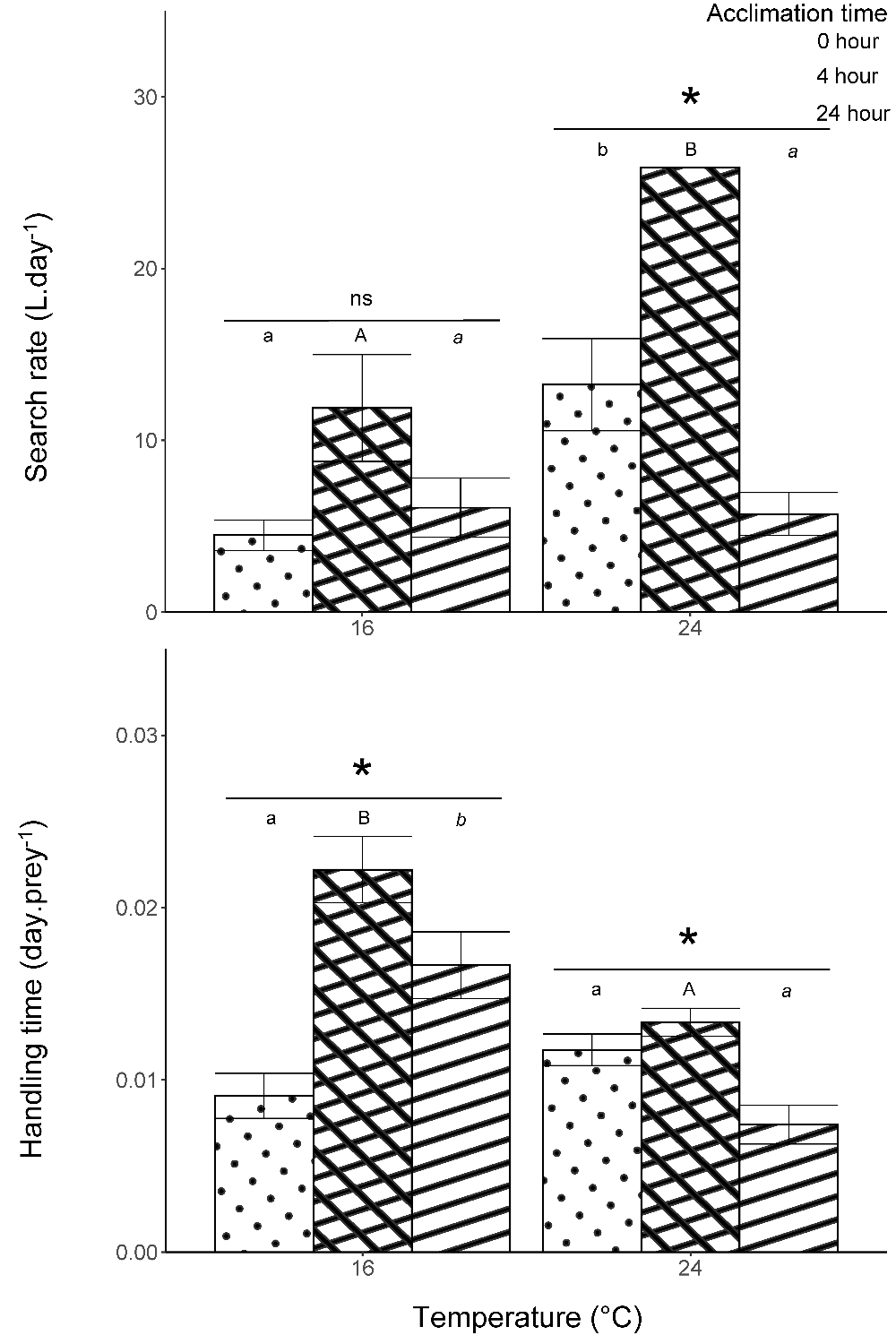


**Figure S3.** Estimated values (mean ± 95% CI) of search rate and handling time for *Procambarus virginalis* feeding on *Asellus aquaticus* for different temperatures and acclimation times. Parameters were estimated using the number of prey eaten instead of the total number of prey killed. Significant differences (p < 0.05) between temperatures marked by asterisk. Different letters denote significant differences within acclimation time between temperatures (0 hours = small letter, 4 hours = capital letter, 24 hours = small letter in italic). Predators and prey were maintained at 20°C before thermal acclimation started.
